# Discovery of High-Affinity Protein Binding Ligands – Backwards

**DOI:** 10.1371/journal.pone.0010728

**Published:** 2010-05-19

**Authors:** Chris W. Diehnelt, Miti Shah, Nidhi Gupta, Paul E. Belcher, Matthew P. Greving, Phillip Stafford, Stephen Albert Johnston

**Affiliations:** 1 Center for Innovations in Medicine, Arizona State University, Tempe, Arizona, United States of America; 2 Center for BioOptical Nanotechnology, The Biodesign Institute, Arizona State University, Tempe, Arizona, United States of America; 3 School of Life Sciences, Arizona State University, Tempe, Arizona, United States of America; Tulane University Health Sciences Center, United States of America

## Abstract

**Background:**

There is a pressing need for high-affinity protein binding ligands for all proteins in the human and other proteomes. Numerous groups are working to develop protein binding ligands but most approaches develop ligands using the same strategy in which a large library of structured ligands is screened against a protein target to identify a high-affinity ligand for the target. While this methodology generates high-affinity ligands for the target, it is generally an iterative process that can be difficult to adapt for the generation of ligands for large numbers of proteins.

**Methodology/Principal Findings:**

We have developed a class of peptide-based protein ligands, called synbodies, which allow this process to be run backwards – i.e. make a synbody and then screen it against a library of proteins to discover the target. By screening a synbody against an array of 8,000 human proteins, we can identify which protein in the library binds the synbody with high affinity. We used this method to develop a high-affinity synbody that specifically binds AKT1 with a K_d_<5 nM. It was found that the peptides that compose the synbody bind AKT1 with low micromolar affinity, implying that the affinity and specificity is a product of the bivalent interaction of the synbody with AKT1. We developed a synbody for another protein, ABL1 using the same method.

**Conclusions/Significance:**

This method delivered a high-affinity ligand for a target protein in a single discovery step. This is in contrast to other techniques that require subsequent rounds of mutational improvement to yield nanomolar ligands. As this technique is easily scalable, we believe that it could be possible to develop ligands to all the proteins in any proteome using this approach.

## Introduction

For the proteomic revolution to be as comprehensive as the genomic revolution, a large number of protein binding ligands, at least one for each protein, are needed to specifically detect low concentrations of a single protein in the presence of a complex background of proteins, peptides, and lipids [1]. Antibodies are the most widely used ligand, but can be expensive to produce with limited control of the production time or the binding properties for the target protein. These factors have limited the availability of antibodies for large-scale proteomics applications and have motivated numerous efforts to develop antibodies and non-antibody based protein-binding reagents [1], [2], [3], [4], [5], [6].

**Figure 1:**
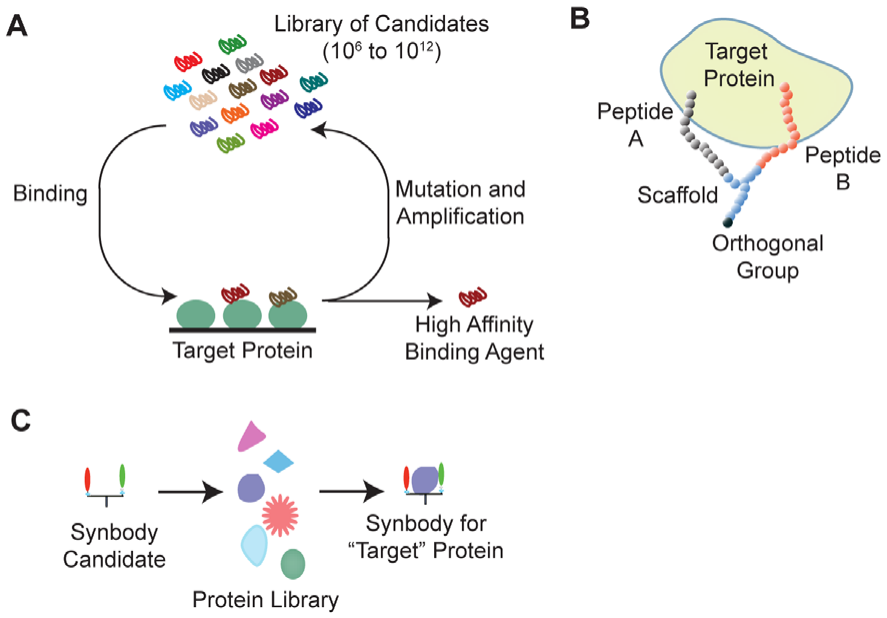
Ligand discovery by iterative selection or by backwards selection. (**A**) Traditional *in vitro* ligand discovery. (**B**) Illustration of a synbody binding to a model protein. Peptide A (gray) and peptide B (red) are synthesized on a peptidic scaffold (blue) that contains an orthogonal functional group (black) that can be used for conjugation to fluorescent dyes, affinity tags, or solid supports. (**C**) Ligand discovery by screening a single ligand against a panel of proteins.

Current systems to produce non-antibody protein-binding reagents use in vitro methods, such as phage and mRNA display, or SELEX to generate high-affinity ligands to one target protein at a time (Figure 1A) [7], [8], [9], [10], [11], [12]. These methods have been very successful in generating affinity reagents by searching large libraries of oligonucleotides, small protein domains, or small peptides, to identify a few reagents with high affinity for the target. However, these are linear methods that can consume large quantities of target protein and can take a significant amount of time due to their iterative nature. It has been noted by the head of the Human Protein Atlas, that no existing system offers the potential for high-throughput (HTP) ligand production [13].

Our solution to the affinity reagent problem is to develop a new class of affinity reagents that can be developed in a high-throughput manner using minimal amounts of protein. These reagents should perform like an antibody yet not be subject to some of the limitations imposed by biological production systems, namely lack of epitope control, long development time and high cost of production. Additionally, the reagent should be chemically synthesized so that a stable supply of reagent can be produced at a reasonable cost. With these requirements in mind, we devised a new protein binding ligand called a synthetic antibody or synbody that is composed of two peptides linked by a scaffold to create a high-affinity binding agent (Figure 1B). We chose to use long, unstructured peptides, 20 amino acids (aa) in length, as previous work has shown that these peptides provide better binding specificity [14]. Two peptides that have low affinity for a target protein are then linked together to improve the binding affinity of the construct, a well-known technique to create high affinity ligands from two low affinity ligands [15], [16], [17], [18], [19]. Finally, an orthogonal functional group is used in the linker to allow the synbody to be coupled to reporter molecules, solid-supports, or to other proteins.

Although synbodies can be readily produced in a linear fashion starting with a specific protein target [20], the process, like producing antibodies, is not high-throughput. In searching for a method to design a high-throughput ligand generation system, we wondered if it were possible to build a synbody and then screen it against a library of proteins to discover its target – in essence by making ligands backwards (Figure 1C). This idea was built on the hypothesis that a long (>15 aa), linear peptide has a high probability of binding, P_pep1_, at least weakly to a surface on one or more proteins in a proteome. By linking together two linear peptides to create a synbody, the probability that a synbody would bind a protein, P_Syn_, would be less than or equal to the product of the probabilities of binding of each peptide, assuming each peptide binds an independent site on the target protein.
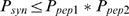
If this probability was high and a large library of proteins were sampled, N_Sampled_, then it is should be possible to screen a single synbody and discover a number of protein targets, N_Expected_, for that synbody.
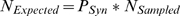
The availability of arrays of large number of proteins makes this concept testable and the system potentially high-throughput.

## Results and Discussion

**Figure 2:**
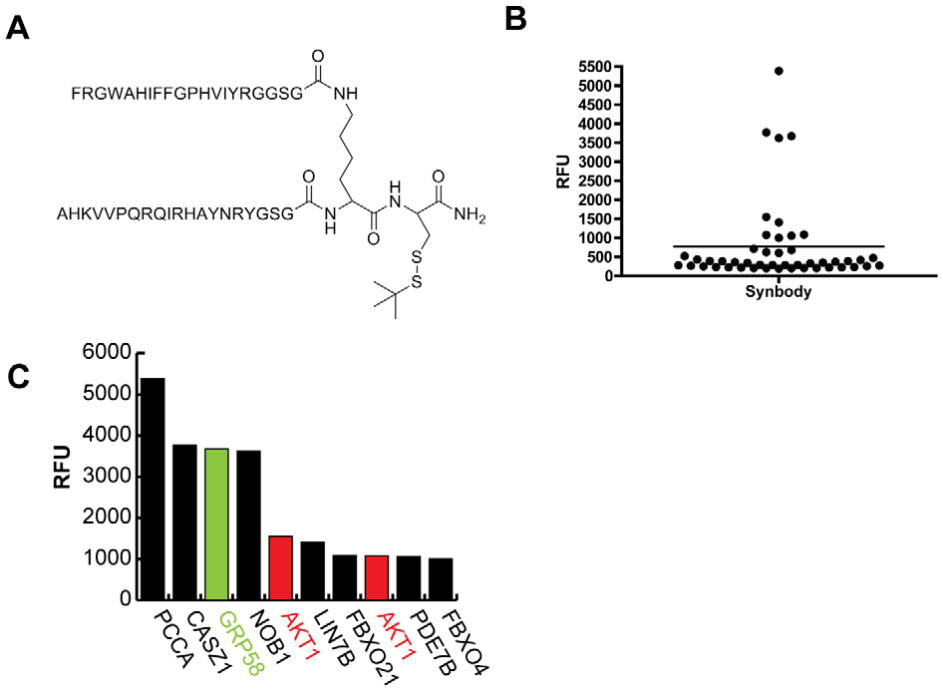
Synbody binding to proteins on protein array. (**A**) Synbody construct screened against 8,000 protein array. (**B**) Scatter plot of synbody binding to top 50 proteins on protein array. (**C**) Relative fluorescence observed for the top 10 proteins bound by the synbody.

To test this approach, a candidate synbody was constructed using two peptides, AHKVVPQRQIRHAYNRYGSG and FRGWAHIFFGPHVIYRGGSG, referred to as peptides 1 and 2, respectively. The sequences of the first 17 aa of these peptides were generated using a random number generator and three constant positions on the C-terminus that served as a spacer between the linker scaffold and the active portion of each peptide. A divergent synthetic method was used in which peptide 1 was synthesized from C to N terminus from the α-amine of lysine and peptide 2 was synthesized from the ε-amine of lysine (Figure 2A, Materials and Methods S1, Figure S1). The synbody was tagged on the C-terminus with biotin and screened against a Life Technologies ProtoArray™. ProtoArrays are nitrocellulose coated slides onto which 8,303 recombinant human proteins have been spotted. After a 2-hour incubation, the array was washed and bound synbody was detected using fluorescently labeled streptavidin. The synbody bound few proteins on the array as seen in the distribution plot of the top 50 background-subtracted spots (Figure 2B). Analysis of the brightest spots revealed that the synbody bound several different proteins including PCCA, CASZ1, GRP58, NOB1, and AKT1 (Figure 2C). It should be noted that AKT1, FBXO21, PDE7B, and FBXO4 have multiple variants (full-length, partial length, or transcript variants) present on the array, hence the appearance of the protein kinase, RAC-alpha serine/threonine protein kinase (AKT1) twice in the top 10 proteins bound by the synbody. It is likely that some of the proteins bound might be false positives as binding levels on protein arrays can be highly variable, thought to be caused by partial to complete denaturation of the immobilized protein that arises from protein printing and array storage [21]. We choose two proteins, AKT1 and protein disulfide-isomerase A3 (GRP58), for further analysis as recombinant proteins are commercially available.

**Figure 3:**
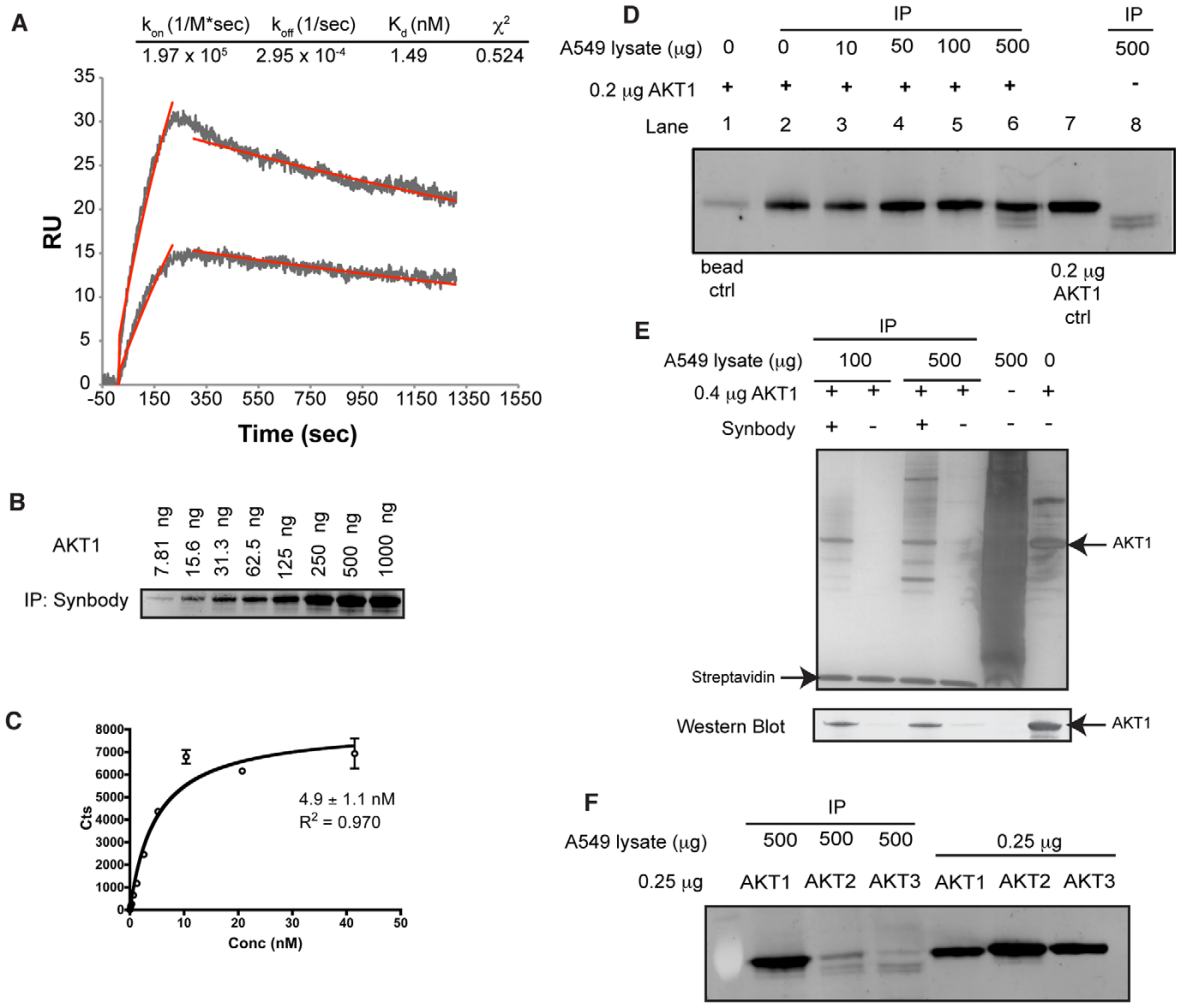
Characterization of synbody binding to AKT1. (**A**) SPR sesnorgrams from 12.5 and 6.25 nM solutions of synbody injected over AKT1. Kinetic fit of the data using a 1∶1 binding model is shown in red. (**B**) IP of AKT1 using the synbody. (**C**) Plot of the amount of ^35^S-labeled AKT1 precipitated by the synbody. A fit of the data with a 1∶1 binding model is shown in black. (**D**) Western Blot detection of 200 ng of AKT1 precipitated in the presence of increasing amounts of A549 cell lysate. Lane 1 is the negative control that shows the amount of AKT1 bound to the streptavidin coated beads from the 200 ng solution of AKT1 in PBS. Lane 8 is the AKT1 control that shows the Western Blot signal from 200 ng of AKT1. (**E**) Silver stain of proteins precipitated by synbody from solutions that contained 400 ng AKT1 spiked into either 100 or 500 µg of pre-cleared A549 cell lysate. The Western Blot confirms that the dominant band precipitated by the synbody was AKT1. (**F**) Western Blot detection from IP of 250 ng of AKT1, AKT2, and AKT3 spiked into 500 µg of A549 cell lysate. The same detection antibody was used for AKT1, AKT2, and AKT3 as it recognized all three forms of AKT.

The synbody (100nM concentration) was screened by Surface Plasmon Resonance (SPR) against immobilized AKT1 and GRP58 on a Biacore T-100 SPR. The synbody appeared to have high affinity for AKT1 and low affinity for GRP58 (Figure S2). To accurately determine the binding kinetics of the synbody-AKT1 complex, several concentrations of synbody were injected over a low-density surface of biotin-labeled AKT1 captured on a Neutratvidin SPR chip using a Biacore A-100. The synbody bound AKT1 and the resulting sensorgram was fit with a 1∶1 binding model to reveal that the synbody had a rapid association rate, k_a_ = 2.0×10^5^ M^−1^*sec^−1^ and a slow dissociation rate, k_d_ = 3.0×10^−4^ sec^−1^ for a dissociation constant, K_d_ = 1.5 nM (Figure 3A). A residual plot was constructed from the difference between the measured sensorgram and the 1∶1 fit of the data at each point in time and the residuals were randomly distributed around zero, which indicated that the 1∶1 binding model was an appropriate model. The synbody's binding affinity is in the same range as that of a monoclonal antibody binding its cognate antigen [22], [23] but was achieved in a single screening step with no further evolution to achieve nanomolar binding affinity.

The synbody was tested for its ability to bind native protein in solution. A variant of the synbody was prepared in which the C-terminal Cys was replaced with a Lys-Biotin. The synbody was used in an immunoprecipitation (IP) experiment in which it was bound to streptavidin magnetic beads and incubated overnight with 89 nM to 0.7 nM solutions of AKT1. The beads were washed extensively and the bound protein was eluted. Samples were run on a SDS-PAGE gel and detected by Western Blot with an anti-AKT1 monoclonal antibody (Figure 3B). As can be seen, the synbody bound AKT1 from solution.

In order to validate the K_d_ determined by SPR, an additional IP was performed using ^35^S-labeled AKT1 produced by in vitro translation (IVT). The translation mixture contained 12 µg of AKT1 in the presence of 400 µg of total protein and a concentration series of ^35^S-labeled AKT1 from 41.5 nM to 80 pM was prepared by dilution of the 35-S-labeled AKT1 IVT mixture. The synbody was then used to precipitate the ^35^S-labeled AKT1 in the same manner as before. Bound AKT1 was quantified using liquid scintillation counting and plotted as a function of AKT1 concentration. The resulting isotherm was fit to a 1∶1 binding model using GraphPad Prism and showed the synbody had a K_d_ of 4.9±1.1 nM, in agreement with the SPR results (Figure 3C). It should be noted that this experiment was performed in the presence of a cell lysate in which AKT1 was approximately 3% of the total protein in solution, indicating that the synbody specifically binds AKT1 from a complex background.

As a preliminary test of specificity, 200 ng of AKT1 (33.2 nM) was spiked into increasing amounts of cell lysate prepared from un-stimulated A549 lung epithelial cells and streptavidin beads coated with 25 pmols of synbody (7.6∶1 synbody-to-AKT1 ratio) were used to pull down AKT1 using the same IP procedure and Western Blot protocol as before. As can be seen in Figure 3D, the synbody successfully pulled AKT1 out of the cell lysate in the presence of an increasing concentration of A549 lysate with little reduction in AKT1 signal intensity. This result indicates that the synbody has sufficient affinity and specificity for AKT1 to precipitate it in the presence of 2,500 fold excess cell extract. Note that in lane 6 at higher A549 lysate concentration, the synbody precipitates the very low level endogenous AKT1 from the un-stimulated cell lysate. This result was confirmed in lane 8 where the synbody was used to precipitate AKT1 from the un-stimulated A549 lysate when no recombinant AKT1 was present.

However, to more rigorously test the specificity of the synbody, we reduced the molar ratio of synbody to protein to approximately 2 to 1 and performed an IP to examine other proteins that were non-specifically bound by the synbody. In this experiment, 400 ng of AKT1 (66.4 nM) was spiked into 100 and 500 µg of A549 cell lysate pre-cleared on streptavidin beads (Figure S3) and precipitated using streptavidin beads coated with 15 pmols of synbody. Synbody coated beads and uncoated streptavidin beads were incubated overnight, washed, and bound proteins were eluted. Elution samples were split with half of the sample analyzed by silver stain and half analyzed by Western Blot (Figure 3E). From the silver stained gel, it can be seen AKT1 was precipitated along with two other prominent proteins from the 500 µg (5mg/ml) sample. This result indicates the synbody has useful, though not perfect specificity.

When the synbody was screened on the ProtoArray, it showed little binding to AKT2, or AKT3 on the array, which share 92% and 87% sequence identity with AKT1, respectively. This apparent specificity was investigated further by performing an IP using 250 ng of each isoform of AKT spiked into 500 µg of A-549 cell lysate. As can be seen in Figure 3F, the synbody selectively precipitates AKT1 while precipitating relatively little AKT2 or AKT3, which suggests that the synbody makes unique contacts on AKT1 that are not present on either AKT2 or AKT3. This result is in contrast to an anti-AKT1 monoclonal antibody that recognized all three AKT variants on the protein array (Figure S4).

The simplicity in creating this high affinity ligand to AKT1 was based on the hypothesis that each unstructured peptide bound a different site on AKT1 and that the synbody affinity was driven by the product of the two peptide's affinity [16], [17], [18], [19], [20]. However, it is possible that the high-affinity binding of just one of the peptides drives the synbody affinity. To test if the binding was driven by a single peptide, we used SPR to screen the individual peptides that made up each arm of the synbody against immobilized AKT1. Each peptide was synthesized on an automated peptide synthesizer and purified to >95% purity by HPLC with confirmation by MALDI-TOF-MS. Each peptide was analyzed by SPR against immobilized AKT1 and the individual peptides bound AKT1 with micromolar affinity (K_d_∼2 to 20 µM). Representative sensorgrams are shown in Figure S5 and are indicative of a low affinity interaction between the peptides and AKT1. These data imply that the high affinity exhibited by the synbody is not simply driven by a single high affinity peptide but could be the consequence of a bivalent interaction between each peptide and AKT1.

**Figure 4:**
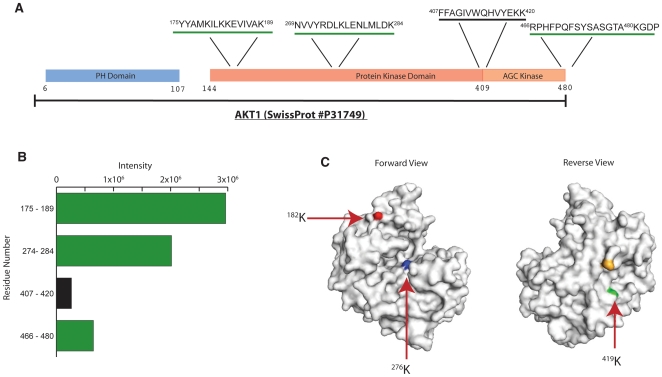
The two component peptides of bind different sites on AKT1. (**A**) Sequence of AKT1 illustrating three different domains and the peptides that were identified by crosslinking. Peptides that crosslinked to peptide 1 are indicated by the black underline while those crosslinked to peptide 2 are underlined in green. (**B**) Bar chart of mass spectral intensity (counts) of each cross-linked peptide. Black bars correspond to fragments crosslinked to peptide 1 and green bars to fragments crosslinked to peptide 2. (**C**) Location of surface exposed lysine's from each crosslinked fragment mapped onto the crystal structure of residues 139–480 of AKT1 (PDB# - 3CQU).

As an orthogonal assessment of whether the peptides bind different sites on AKT1, each peptide was crosslinked to AKT1 *in vitro* using a commercially available set of deuterated / non-deuterated crosslinkers (BS^3^-(d_0_/d_4_)). The crosslinkers chemically conjugate primary amines that are in close proximity to one another (11.4 Å) and the crosslinked complex is then subjected to enzymatic digestion and analysis by mass spectrometry. Crosslinked peptides are identified by the presence of a pair of ions that are separated by 4 mass units indicating crosslinking with both non-deuterated and deuterated crosslinkers. Peptide 1 has two possible crosslinking sites (N-terminus and K at position 3) and peptide 2 has one possible crosslinking site at the N-terminus. A 10 µM solution of each peptide was added to 2.5 µM AKT1 and incubated for 1 hour prior to the addition of a 100 µM solution of a 1∶1 mixture of crosslinkers. After crosslinking, the mixture was digested with trypsin and analyzed by MALDI-TOF-MS. It was found that peptide 1 crosslinked to a region of AKT1 that spans the Protein Kinase (PK) and AGC Kinase domain while peptide 2 crosslinked to multiple regions on AKT1 (Figure 4A, Figure 4B, Table S1), with the majority of crosslinking occurring in the PK domain, in the region of the ATP binding site (residue 179) and the active site (residue 274). The location of each AKT1 fragment was mapped onto a crystal structure of residues 139–480 of AKT1 (PDB #3CQU) (Figure 4C). As amine reactive cross-linkers will only react with the side chains of surface exposed lysines, we analyzed the identified AKT1 fragments for surface exposed lysines and found that each peptide had 1 exposed lysine. Peptide 1 most likely cross-linked to ^419^Lys given that the side chain is exposed while peptide 2 likely cross-linked to ^182^Lys, ^276^Lys, and ^481^Lys.

The relative intensity data for each peptide 2-AKT1 fragment and the close proximity of ^182^Lys and ^276^Lys, suggests that the primary binding site for peptide 2 is likely on the PK domain and that a secondary binding site lies on the AGC domain. The locations of the cross-linked lysines indicate that it is possible for the N-terminal region of each peptide to bind AKT1 when linked as a bivalent synbody. These data and the micromolar binding affinity of the individual peptides suggest that the high-affinity and high specificity of the synbody is the product of a bivalent interaction. Additionally, as the binding site of peptide 1 is in a region of low homology between AKT1, AKT2, and AKT3 (Figure S6), the selectivity for AKT1 is likely a product of a bivalent interaction.

**Figure 5:**
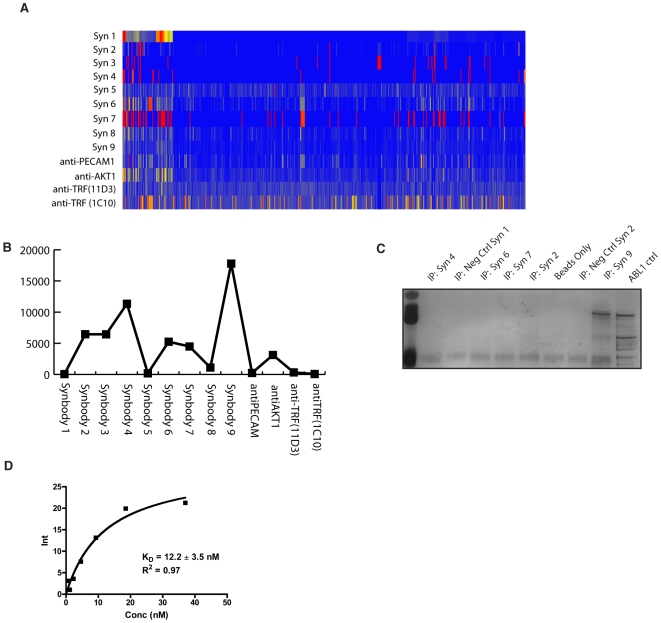
Discovery and characterization of a synbody to ABL1. (**A**) Heat map of median-normalized fluorescence of 9 synbodies and 4 monoclonal antibodies binding to 8,000 proteins from protein array. Each protein on the array is displayed on the x-axis and the binding intensity is indicated by color with red corresponding to high intensity and blue corresponding to no binding. Intensities range from 0.01 (blue) to 35 (red) across all experiments, with yellow indicating intermediate intensity. (**B**) Background subtracted fluorescence of 9 synbodies and monoclonal antibodies binding to ABL1. (**C**) IP screen of ABL1 synbody candidates against a 54 nM solution of ABL1 in PBST. (**D**) Binding isotherm of the amount of ABL1 precipitated by synbody 9. A fit of the data with a 1∶1 binding model is shown in black.

To test if this method to produce synbodies backwards is generally applicable and amenable to HTP parallel processing, 8 other synbodies were synthesized and screened on ProtoArrays (Materials and Methods S1, Figure S7, Figure S8, Figure S9, Table S2). Additionally, we screened 4 monoclonal antibodies to compare the synbody binding profiles to those of high-affinity antibodies. A heat map was constructed from the median normalized fluorescent data and in each case the synbody produced a unique binding profile suggesting that each synbody behaves as a unique binding entity (Figure 5A). Each synbody bound several proteins. It should be noted that monoclonal antibodies often bind several proteins when screened on protein arrays [24], [25], as was the case for the 4 monoclonal antibodies that we screened.

From the heat map, some proteins, such as the proto-oncogene tyrosine kinase ABL1, appeared to bind to multiple synbodies (Figure 5B). We performed an additional screen of 5 synbodies that bound ABL1 on the protein array plus 2 negative control synbodies that were not run on the ProtoArray to test if the backwards method could be used to identify a functional synbody to ABL1. We purchased recombinant full-length ABL1 and used the same IP protocol as before to precipitate 1µg of ABL1 (∼700 ng of full-length ABL1 for a concentration of ∼54 nM) in a phosphate buffered saline solution. The silver stained gel showed that only synbody 9 pulled down ABL1 (Figure 5C). Subsequent IP assays that used a concentration gradient of ABL1 demonstrated that synbody 9 had high affinity for ABL1, K_d_ = 12 nM (Figure 5D). We tested the specificity of synbody 9 for ABL1 in a pull-down assay in which 800 ng of ABL1 (though by western blot only a small portion of this was full-length protein) was spiked into 100 or 500 µg of pre-cleared A549 lysate (1 mg/mL and 5 mg/mL protein concentration) and precipitated with synbody 9. Fifteen pmols of biotinylated synbody 9 was captured on streptavidin coated beads (∼3∶1 synbody-to-ABL1 ratio) and the proteins precipitated by the synbody were analyzed by silver stain and Western Blot (Figure S10). The synbody precipitated full-length ABL1 in the presence of >150 and >850 fold excess protein. Note that the synbody only appears to bind full-length protein. The Western blot indicates the synbody also precipitated one other prominent protein from the extract. These results demonstrate that a high-affinity, moderate specificity synbody can be readily generated to another protein using this procedure.

Generating ligands to the human and other proteomes is a major challenge. The unique features of synbodies in combination with protein arrays may offer a solution. Synbodies are synthesized by standard peptide chemistry, enabling a large number of them to be produced in parallel. The ligand screening step is rapid and can be run in parallel, but the major cost in this system as demonstrated is the protein arrays. This limitation could be overcome by multiplexing the synbodies on each array or using other protein array technologies [21], [26], [27]. It should be noted that these synbodies were generated against native protein. While we have found that they do function in Western blots, synbodies optimized for functioning in Western blots could be isolated in screens against denatured proteins.

The two initial synbodies discovered by this method demonstrated binding affinities in the range of antibodies with specificities approaching those of commercial antibodies. These synbodies have low sequence diversity, as synbody 9 and synbody 1 share the same stretch of 20 amino acids; yet have completely different protein targets. This echoes recent work that has shown that low complexity ligand libraries that are highly biased for Tyr and Ser can be used to produce high affinity and high specificity ligands for a variety of protein targets [28], [29], [30]. By incorporating these findings into an improved design of the peptides that make up the synbody, it should be possible to improve the binding affinity and binding specificity. Additionally, we are developing an affinity optimization method for the peptide arms of the synbody (M. Greving, N. Woodbury, P.E.B, C.W.D and S.A.J., unpublished results) that could also be used to improve affinity and specificity. With these improvements to the first generation of synbodies, we believe that the backward process of ligand generation could offer the possibility of creating binding agents to the human proteome.

## Materials and Methods

### Reagents and materials

Recombinant human AKT1 was purchased from Life Technologies (Carlsbad, CA, USA) while GRP58 was purchased from Abnova (Taiwan). Recombinant AKT2 and AKT3 were purchased from Millipore (Milford, MA, USA). Recombinant ABL1 was purchased from Abcam and only 70% pure as stated by the manufacturer. ABL1 concentrations are adjusted to reflect this. The anti-ABL1 polyclonal antibody was purchased from Abcam (Cambridge, MA, USA). Monoclonal antibodies for transferrin (TRF) were purchased from Abcam. The anti-AKT1 antibody was purchased from Millipore and cross-reacts with AKT2 and AKT3. The anti-PECAM1 monoclonal antibody was supplied as a gift from Life Technologies (Carlsbad, CA, USA).

### Synbody synthesis

Synbodies were synthesized using two different approaches: i) multiple antigenic peptide (MAP) [31] constructs were synthesized using a modified divergent Fmoc solid phase synthesis using orthogonal protecting groups on branched lysine; ii) linear synbodies were synthesized using target peptides that were modified with either an azido or alkyne group and linked using a Click reaction [32] to either (Pro-Gly-Pro) [33] or (Pro-Pro-Pro)_6_ [34] scaffold. Details of each synthetic approach are described in Materials and Methods S1.

All reagents and solvents were analytical, HPLC or peptide synthesis grade. Commercial reagents and solvents were obtained from Aldrich and Fisher respectively and used without further purification unless otherwise noted. All amino acids and resins were purchased from Novabiochem, Chem Impex International Inc. as well as from Advanced Chem Tech and used without further purification. All peptides were synthesized via standard Fmoc stepwise solid phase peptide synthesis (SPPS) on a Symphony Multiple Peptide Synthesizer at 25umole scale. Matrix-assisted laser desorption/ionization time-of-flight mass spectrometry (MALDI-TOF/MS) was carried out on a Bruker Microflex. UV measurements were carried out on a ND-1000 spectrophotometer instrument. All reversed-phase HPLC analysis and purifications were conducted on an Agilent 1200. Phenomenex Luna 5u analytical (4.6×250 mm) and semi-preparative (10×250 mm) C-18 columns were used for the analysis and purification.

### Protein Array experiments

Synbodies were screened on an Invitrogen ProtoArray™ (Life Technologies, Carlsbad, CA, USA) detected with Alexa-555 labeled streptavidin (Life Technologies), and scanned using a Perkin Elmer ProScan Array HTP. The spots were analyzed using GenePix Pro (Molecular Devices, Sunnyvale, CA, USA) and data were processed using Genespring GX (Agilent Technologies, Santa Clara, CA, USA). Synbodies were labeled with biotin using either Maleimide-PEG_2_-Biotin (Thermo Pierce, Rockford, IL, USA) to label the C-terminal cysteine or biotin was incorporated during synbody synthesis by replacing the C-terminal cysteine with Lys-biotin (Novabiochem, San Diego, CA, USA). Synbodies were screened on the array using the recommended protocol with the following modifications: the arrays were blocked with 5 mL blocking buffer (50mM HEPES pH 7.5, 200 mM NaCl, 0.08% Triton X-100, 25% Glycerol, 20mM reduced Glutathione, 1.0mM DTT, 1% BSA) for 90 min. A 200 nM synbody solution was incubated in probing buffer (1× PBS, 1% BSA, 0.1% Tween) for 90 minutes. Slides were dried by centrifugation at 200g for 1 minute and scanned at 100% laser power and 70% photomultiplier voltage. The gal file was obtained for each array by submitting the barcode of the array to www.invitrogen.com/protoarray.

### Data Analysis

Heat maps were constructed using median-normalized data from each ProtoArray. All responses from the Protoarray were included except for spots flagged “poor” in GenePix. This includes spots that failed Invitrogen's recommended quality control specifications. Hierarchical clustering using Euclidean distance was performed on synbody 1 data so that other experiments can be compared directly to a single ProtoArray intensity.

### Synbody Screening by SPR

SPR screening of the synbody against AKT1 and GRP58 was performed on a Biacore T-100 (GE Healthcare, Piscataway, NJ, USA). The series S sensor chip CM5, amine coupling reagents and HBS-EP were obtained from GE Healthcare. A standard amine immobilization protocol was performed at 25°C using NaHCO_3_ (10mM in 150mM NaCl, pH 4.5) as the immobilization buffer. The CM-5 dextran chip was activated by a 10 minute injection of a freshly prepared 1∶1 solution of 400 mM 1-ethyl-3-(3-dimethylaminopropyl)-carbodimide (EDC): 100 mM N-hydroxysuccimide (NHS) in water. The NHS-carboxy groups of the dextran surface were treated with a solution of AKT1 or GRP58 (25 µg/ml) in sodium acetate (pH 5.0) for 8 mins at a flow rate of 10 µl/min. Any residual active sites were then quenched by a 5 min pulse of ethylene diamine (1M, pH 8.5). A 100 nM solution of the synbody was injected at 30 µl/min across each protein for a 90 second association phase and a 240 second dissociation phase. Data were processed using the software supplied by Biacore.

### Biotinylation of AKT1

To a solution of AKT1 (25µg, 4.16×10^−10^ moles, 1.0 equiv.) in 50mM Na_2_CO_3_ in 150mM NaCl, pH 9.0 (200 µl), was added a solution of NHS-LC-LC-Biotin (Thermo Scientific) (9.4 µl, 8.32×10^−10^ moles, 2.0 equiv.) in DMSO. The reaction was kept on ice at 0°C with frequent agitation for 2 hours, before the mixture was transferred to a 10 kDa Millipore Microcon filter and diluted with HBS-N (200 µl). The excess biotin was removed by spinning the filter at 12,000 G until the volume on top of the filter had been reduced to 0.25 the original volume. The retentate was washed with an additional 200 µl of HBS-N and spun for a further 15 minutes. The filter was then flipped over and the protein recovered (15 µg, 60%). After filtration, an appropriate volume of 10× HBS-N was added to the sample to increase the HBS-N concentration to a 1× equivalent.

### SPR determination of K_d_ for AKT1 synbody

Determination of the binding kinetics of the synbody on AKT1 was performed on a Biacore A-100. The series S sensor chip CM5, amine coupling reagents and HBS-EP were obtained from GE Healthcare. A standard amine immobilization protocol was performed at 25°C using NaHCO_3_ (10mM in 150mM NaCl, pH 4.5) as the immobilization buffer. Spots 1,2 and 3 on each of the four flow cells of the CM-5 dextran chip were activated by a 10 minute injection of a freshly prepared 1∶1 solution of 40 0mM EDC : 100 mM NHS in water. The NHS-carboxy groups of spot 1 of the dextran surface were treated with a solution of neutravidin (50 µg/ml) in sodium acetate (pH 5.0) for 8 mins at a flow rate of 10 µl/min. Any residual active sites were then quenched by a 5 min pulse of ethylene diamine (1M, pH 8.5). Similarly neutravidin was coupled to spots 2 and 3 via a 12 and 16 min injection cycle respectively, followed by quenching. An additionally 7 min injection of ethylene diamine was then pulsed over spots 1–3 to ensure no activated sites remained. This immobilization process was then repeated to couple neutravidin spots 4 and 5.

Prior to capture of the affinity tagged protein, the chip surface was conditioned with 8–30 second pulses of 10 mM hydrochloric acid. Biotinylated AKT1 was injected at 10 µL/min until the desired level of capture was achieved. Two consecutive 30-second pulses of amino-PEG_2_-Biotin (1mM in HBS-N) were used to quench any excess biotin binding capacity of neutravidin.

For high-resolution kinetics, biotin-tagged AKT1 was captured in multiple spot densities with Rmax's ranging from 20–100 RUs. Synbody samples spanning 25 nM to 0.78 nM in concentration were injected in a random order at a flow rate of 30 µl/min over the flow cell surface. Buffer injections identical to the analyte were included throughout the analysis for the purpose of double referencing. Association and dissociation were monitored for 3 and 16 min, respectively. The surfaces were regenerated with 1 30-s injection of pH 2.5 glycine. To determine the kinetic parameters of the interaction, each data set was double referenced and fit globally using a 1∶1 interaction model using Biacore A100 Evaluation or BiaEvaluation Software.

### Production of ^35^S-labeled AKT1

AKT1 DNA was assembled by ligation of 42-bp oligonucleotides by PCR according to a procedure developed internally (Borovkov, et al. submitted). The PCR product was cloned into pEXP5-NT topo expression vector (Life Technologies), purified using a Qaigen PCR purification kit, and quantified by Nanodrop spectrophotometer (Thermo Scientific). Approximately 1µg of the plasmid containing ATK1 gene was used for in vitro translation (IVT) using the Expressway™ Mini Cell-Free Expression System (Life Technologies). The protein synthesis was carried out in the ProteoMaster incubator following the manufacturers protocols. After the reaction was complete, it was placed on ice to stop the reaction. Approximately 5 µL of supernatant was spotted on a glass fiber filter for TCA precipitation and yield determination. For imaging, 5 µL of the IVT reaction was loaded on the SDS acrylamide gel (Bio-Rad Precast Criterion XT 26-well 4%–12% Bis-Tris). Gel was run at 150V for 55 minutes, stained with SimplyBlue (Life Technologies) and dried under vacuum. Radioactive labeled proteins were transferred to a phosphor screen and visualized on a Typhoon Trio.

### Immunoprecipitation experiments

For all IP experiments, either a 1 µM or 250 nM solution of biotin-conjugated synbody was captured on 20 µL of streptavidin coated paramagnetic M-280 Dynabeads (Life Technologies) according to the manufacturers protocol. Synbody coated beads were washed five times with 1 mL of 100 mM phosphate buffered saline containing 0.05% Tween-20 (PBST) and incubated overnight at 4°C with shaking in the desired amount of target protein in A549 cell lysate. For K_d_ determination experiments, serial dilutions of AKT1 or ABL1 were prepared in binding buffer. After binding, beads were washed five times with 1 mL of PBST and heated at 70°C in 20 µL of 1× LDS loading buffer (Invitrogen) for 10 minutes. The samples were analyzed by SDS-PAGE gel with Western Blot detection using the appropriate antibody and imaged on a GE Healthcare Typhoon Trio.

### AKT1 Binding Site Mapping

10 µM of peptide 1 and peptide 2 were individually incubated with 2.5 µM of recombinant AKT1 (produced in house) in PBS buffer (200 uL total volume) for one hour, after which an equimolar ratio of BS^3^-d_0_∶BS^3^-d_4_ (Thermo Scientific, Rockford, IL) crosslinker was added for a total crosslinker concentration of 100 µM. The same concentration of crosslinker was added to a 200 µL control sample containing only 2.5 µM AKT1. Crosslinking was performed for one hour. After crosslinking, free peptide and crosslinker were removed from the mixture with a 30 kDa spin filter and at the same time the buffer was exchanged with 100 mM ammonium bicarbonate pH 8.0. Proteomics grade trypsin (Sigma-Aldrich, St. Louis, MO) was added to all samples at a ratio of 1∶30 (mol∶mol) Trypsin∶AKT1 and incubated overnight at 37°C. Digested samples were lyophilized and reconstituted in 5 µL acetonitrile∶H_2_O (1∶1) containing 0.1% trifluoroacetic acid and spotted on a MALDI target plate along with ∝-cyano-4-hydroxycinnamic acid MALDI matrix. Mass spectra were obtained on an Ultraflex-III MALDI TOF-TOF (Bruker, Billerica, MA) and analyzed in FlexAnalysis (Bruker, Billerica, MA).

### IP with Silver Stain Detection

Due to the high-level of A549 lysate protein binding to the magnetic beads used previously, we pre-cleared the cell lysate prior to IP. This was accomplished by overnight incubation of 1 mL of lysate (∼5 mg/mL) with 1 mL of streptavidin beads. The supernatant was removed and this lysate was used in subsequent experiments. The IP experiments were performed as before with the exception that 40 µL of synbody coated beads were used and the beads were washed seven times with PBST prior to elution. One half of each elution sample was analyzed by SDS-PAGE followed by staining using a SNAP Silver Stain kit (Thermo Pierce) while the other half of the sample was analyzed by SDS-PAGE with Western Blot detection as before.

## Supporting Information

Materials and Methods S1 Contains details of synbody synthesis.
(0.05 MB DOC)

Table S1 Table of fragments identified from crosslinking peptide 1 and peptide 2 with AKT1.
(0.24 MB PDF)

Table S2 List of synbodies screened on protein arrays and their amino acid sequence.
(0.25 MB PDF)

Figure S1 Synthesis scheme for MAP synbodies.
(0.22 MB TIF)

Figure S2 SPR sensorgram from injection of 100 nM synbody over A) immobilized AKT1 and B) immobilized GRP58.
(0.35 MB TIF)

Figure S3 Silver stain gel of proteins present in A549 cell lysate that was pre-cleared using streptavidin coated magnetic beads and in un-cleared A549 cell lysate.
(2.68 MB TIF)

Figure S4 Top 10 proteins bound by anti-AKT1 monoclonal antibody on protein array.
(0.09 MB TIF)

Figure S5 Sensorgrams from A) 1.88 µM, 0.94 µM, and 0.47 µM solutions of peptide 1 flowed over 15,584 RU of immoblized AKT1 and B) 20 µM, 10 µM, and 1 µM solutions of peptide 2 over 19,912 RU of immobilized AKT1. Dissociation constants were not determined due to the high immobilization levels of AKT1 used.
(0.95 MB TIF)

Figure S6 ClustalW alignment of AKT1, AKT2, and AKT3 illustrating AKT1 peptides identified in crosslinking experiments.
(2.68 MB TIF)

Figure S7 Synthesis scheme for azido modification of peptides.
(0.21 MB TIF)

Figure S8 Synthesis scheme for alkyne modification of peptides.
(0.13 MB TIF)

Figure S9 Coupling of peptides using CLICK reaction.
(0.22 MB TIF)

Figure S10 Silver stain of proteins precipitated by synbody 9 from solutions that contained 800 ng ABL1 spiked into either 100 or 500 µg of pre-cleared A549 cell lysate. Western Blot of same samples using a polyclonal anti-ABL1 antibody confirming the presence of ABL1.
(4.93 MB TIF)
