# Supplementary material for: Discovery of High-Affinity Protein Binding Ligands – Backwards
Source: PLoS One. 2010 May 19;5(5):e10728. doi: 10.1371/journal.pone.0010728 (PMC2873402; doi:10.1371/journal.pone.0010728)
Supplement: Materials and Methods S1 — Contains details of synbody synthesis. (0.05 MB DOC) [file pone.0010728.s001.doc]

## Supporting Materials and Methods S1

**Abbreviations:** N,N-Dimethylformamide (DMF), Acetonitrile (AcCN), Methyl alcohol (MeOH), Dichloromethane (DCM), 1-Hydroxybenzotriazole (HOBt), 2-(1-H-benzotroazole-1-yl)-1,3,3-tetramethyluronium Hexafluorophosphate (HBTU), N-methylmorpholine (NMM), Trifluoroacetic acid (TFA), N,N-Diisopropylethylamine (DIPEA), Triisopropylsilane (TIPS), 3,6-Dioxa-1,8-octane-dithiol (DoDt), 1-(4,4-Dimethyl-2,6-dioxo-cyclohexylidene)-3-methyl-butyl (ivDde), Fluorenylmethoxycarbonyl (Fmoc), Kaiser reagents (1. Ninhydrine solution; 6% in ethanol, 2. Potassium cynide in pyridine, 3. Phenol in 80% ethanol).

**Synthesis of bivalent Multiple Antigenic Peptides (MAP’s).** MAP synbodies were synthesized via modified divergent Fmoc solid phase peptide synthesis using orthogonal protecting groups on branched lysine. Two orthogonal groups were introduced using Fmoc-Lys(ivDde)-OH at the very C-terminus. The synthesis was carried out at 25 umole scale on Rink amide resin (0.7 mmole/g). The general strategy followed for the synthesis of MAP constructs is shown in Figure S1.

Following removal of Fmoc-protecting group by 20% piperidine in DMF for 5+15 mins, peptide sequence 1 was synthesized on α-amino group of Lysine through stepwise addition of Fmoc amino acids. N-terminus Fmoc group was substituted with Boc group manually by treating with 5 fold excess of (Boc)_2_O (125 umol, 0.027 g) in presence of 10x DIPEA (250 umol, 2.6 nL). The resin was agitated at room temperature for 1 hr followed by standard washings with DMF (3x, 1min each), MeOH (2x, 1min each), DCM (2x, 1min each), DMF (3x, 1min each). An aliquot of resin was taken after MeOH wash for qualitative Kaiser Test. At this point, Nε-(ivDde) protecting group was deprotected manually using 5% hydrazine monohydrate in DMF followed by standard washings. Removal of (ivDde) was monitored spectrophotometrically by absorption of the resulting 3,6,6-trimethyl-4-oxo-4,5,6,7-tetrahydra-1H-indazole at 300nm and was completed in 2+2hrs. Deprotection was also was determined by standard qualitative Kaiser Test.

The stepwise assembly of the peptide sequence 2 was then accomplished at N∑-lysine position again on the peptide synthesizer. A five fold molar excess of Fmoc-amino acids, HOBt and NMM was used throughout the synthesis in a stepwise manner. The final protected MAP was treated with cleavage cocktail (TFA: phenol: H_2_O: TIPS: 90:3:5:2) for 2 hrs at room temperature and precipitated in cold diethyl ether. The precipitated construct was cooled for 15 minutes in -80°C refrigerator to ensure complete precipitation. The solid was separated from the diethyl ether by centrifugation and the top phase was decanted off and pellet re-suspended with another addition of dry diethyl ether. The cooling and centrifugation process was done in triplicate. Upon completion, the construct was dried and dissolved in water for HPLC purification. The construct was purified on reverse-phase HPLC on Phenomenex Luna 5u semi-preparative (10 x 250 mm) C-18 column using solvent system A: 0.1 % TFA in H_2_O; solvent B: 90 % CH_3_CN in 0.1 % TFA with a linear gradient method, 0 min, 10% B; 2 min, 10% B; 20 min, 45% B; 25 min, 95% B; 27 min, 95% B; 30 min, 100% B; 33 min, 10% B) with flow rate of 4 mL/min at a wavelength of 280 nm. The fraction were pooled off and analyzed by MALDI-TOF mass spectrometry. The correct fraction was then lyophilized.

**Synthesis of azido modified peptides.** Peptides with (Pro-Gly-Pro) and (Pro-Pro-Pro)_6_ linkers were synthesized at 25 umole scale using Rink amide resin (0.7 mmol) as shown in Figure S7. Peptides were assembled through stepwise addition of Fmoc amino acids using HOBt/HBTU/NMM as activating agents. All Arginines and Valines were double coupled. The peptide assembly was terminated by N-capping with di-t-butyl dicarbonate manually by treating resin with 5 fold excess of (Boc)_2_O (125 umol, 0.027 g), in presence of 10x DIPEA (250 umol, 2.6 nL) for 1 hr. Reaction mixture was then removed by suction followed by standard washings with DMF (3x, 1min each), MeOH (2x, 1min each), DCM (2x, 1min each), DMF (3x, 1min each). An aliquot of resin was taken after MeOH was for qualitative Kaiser Test.

The Nε-(ivDde) protecting group introduced via Fmoc-Lys(ivDde)-OH at the C-terminus was deprotected manually through treatment of 5% hydrazine monohydrate in DMF followed by standard washings. Removal of (ivDde) was monitored spectrophotometrically by absorption of the resulting indazole at 300 nm and was completed in 2hrs. Deprotection was also was determined by standard qualitative Kaiser Test.

4-(Azidomethyl) benzoic acid, synthesized according to protocol from Sakurai et al [30] (125umol, 0.2g) was incorporated at ε-amino group through HOBt:HBTU:DIPEA (1:1:2) (50uL of 0.5M solution of each HOBt and HBTU in DMF; 2.6nL of DIPEA). The resin was agitated for 1.5hr at room temperature. The azido modified peptide with linker is then, dried in vacuum before cleavage. The peptides were cleaved from resin by treatment of TFA in the presence of phenol, TIPS and water as scavengers. The resin was agitated with cleavage cocktail (TFA: phenol: H_2_O: TIPS: 90:3:5:2) at RT for 2 hours and precipitated in cold diethyl ether. The precipitated construct was cooled for 15 minutes at -80°C to ensure complete precipitation. The solid was separated from the diethyl ether by centrifugation and the top phase was decanted off and pellet re-suspended with another addition of dry diethyl ether. Cooling and centrifugation was done in triplicate. Upon completion, the construct was dried and dissolved in water for HPLC purification. The construct was purified on reverse-phase HPLC on Phenomenex Luna 5u semi-preparative (10 x 250 mm) C-18 column using solvent system A: 0.1 % TFA in H_2_O; solvent B: 90 % CH_3_CN in 0.1 % TFA with a linear gradient method, 0 min, 10% B; 2 min, 10% B; 20 min, 45% B; 25 min, 95% B; 27 min, 95% B; 30 min, 100% B; 33 min, 10% B) with flow rate of 4 mL/min at a wavelength of 280 nm. The fractions were pooled and analyzed by MALDI-TOF mass spectrometry. The correct fraction was then lyophilized.

**Synthesis of alkyne modified peptides:** Peptides were synthesized on Rink amide (0.7 mmol/g)/PEGA Rink amide resin (0.35 mmol/g) without linker and functionalized with 4-pentynoic acid (125 umol, 0.1g) in presence of HOBt:HBTU:DIPEA (1:1:2) (50 uL of 0.5 M solution of each HOBt and HBTU in DMF; 2.6 nL of DIPEA) for 1.5 hr. Cleavage and purification is similar to azido peptides. The synthetic strategy is summarized in Figure S8. Coupling with unnatural amino acid Fmoc-L-Propargylglycine during SPPS is another way of introducing alkyne moiety in a peptide sequence. Peptide 2 was synthesized with Fmoc-L-Propargylglycine and used in Cu(I) catalyzed [3+2]cycoadditions.

**CLICK coupling of synbodies.** Synbodies were then assembled using Cu(I) catalyzed Huisgen reaction, shown in Figure S9. Azido modified peptide with linker (0.1 umol) and alkyne-functionalized peptide (0.2 umol) were dissolved in water. To this added, sodium ascorbate (Vc) (1umole, freshly prepared in water) followed by copper (II) sulfate solution (1umol, freshly prepared in water). The reaction mixture was stirred at room temperature for 12 hours. The reaction mixture was purified on reverse-phase HPLC on Phenomenex semi-preparative (10 x 250 mm, Luna 5u) C-18 column using solvent system A: 0.1 % TFA in H_2_O; solvent B: 90 % CH_3_CN in 0.1 % TFA with a linear gradient method, 0 min, 10% B; 2 min, 10% B; 20 min, 45% B; 25 min, 95% B; 27 min, 95% B; 30 min, 100% B; 33 min, 10% B) with flow rate of 4 mL/min at a wavelength of 280 nm. The fractions were pooled off and analyzed by MALDI-TOF mass spectrometry. The correct fraction was then lyophilized.
