# Supplementary material for: Discovery of High-Affinity Protein Binding Ligands – Backwards
Source: PLoS One. 2010 May 19;5(5):e10728. doi: 10.1371/journal.pone.0010728 (PMC2873402; doi:10.1371/journal.pone.0010728)
Supplement: Table S1 — Table of fragments identified from crosslinking peptide 1 and peptide 2 with AKT1. (0.24 MB PDF) [file pone.0010728.s002.pdf]

Residue Number	AKT1 Fragment	Peptide Fragment	Predict. X-link BS_3-d0 (Da)	Predict. X-link BS_3-d4 (Da)	Obs. X-link BS_3-d0 (Da)	Obs. X-link BS_3-d4 (Da)
407 - 420	(R)FFAGIVWQHVYEKK(L)	AHKVVPQR	2824.55	2828.58	2824.52	2828.51
175 - 189	(R)YYAMKILKKEVIVAK(D)	FR	2257.36	2261.36	2257.76	2261.77
175 - 189	(R)YYAMKILKKEVIVAK(D)-Ox	FR	2273.35	2277.35	2273.79	2277.79
274 - 284	(R)DLKLENLMLDKDGHIK(I)	FR	2358.29	2362.29	2358.84	2362.86
466 - 490 (plus KGDP from vector)	(R)RPHFPQFSYSASGTAKGDP(-)	FR	2510.27	2514.27	2510.91	2514.90
