# Supplementary material for: Discovery of High-Affinity Protein Binding Ligands – Backwards
Source: PLoS One. 2010 May 19;5(5):e10728. doi: 10.1371/journal.pone.0010728 (PMC2873402; doi:10.1371/journal.pone.0010728)
Supplement: Table S2 — List of synbodies screened on protein arrays and their amino acid sequence. (0.25 MB PDF) [file pone.0010728.s003.pdf]

Synbody	Sequence
Synbody 1	AHKVVPQRQIRHAYNRYGSG–KC–FRGWAHIFFGPHVIYRGGSG
Synbody 2	AHKVVYQRQIRFAYNRYGSG–KC–FRGWAHIFFGPHVIYRGGSG
Synbody 3	FRGWAHIFFGPHVIYRGGSG–KC–FRGWAHIFFGPHVIYRGGSG
Synbody 4	FERSYLKMPWKFLQSRQGSG–KC–WGPSYKFKITRFHQQSSGSG
Synbody 5	EGEWTEGKLSLRGSG–KC–GTEKGTSGWLKTGSG
Synbody 6	EGWWTEGKLSLRGSG–KC–GTEKGTSGWLKTGSG
Synbody 7	AHKVVPQRQIRHAYNRYGSG–PGPKGK(biotin)G–FRGWAHIFFGPHVIYRGGSGKSG
Synbody 8	AHKVVPQRQIRHAYNRYGSG–$(PPP)_6$KGCG–FRGWAHIFFGPHVIYRGGSGKSG
Synbody 9	AHKVVPQRQIRHAYNRYGSG–PGPKGK(biotin)G–HAYKGPGDMRRFNHSGMxSG
	x = L-propargylglycine
