# Supplementary figures and images for: Discovery of High-Affinity Protein Binding Ligands – Backwards
Source: PLoS One. 2010 May 19;5(5):e10728. doi: 10.1371/journal.pone.0010728 (PMC2873402; doi:10.1371/journal.pone.0010728)

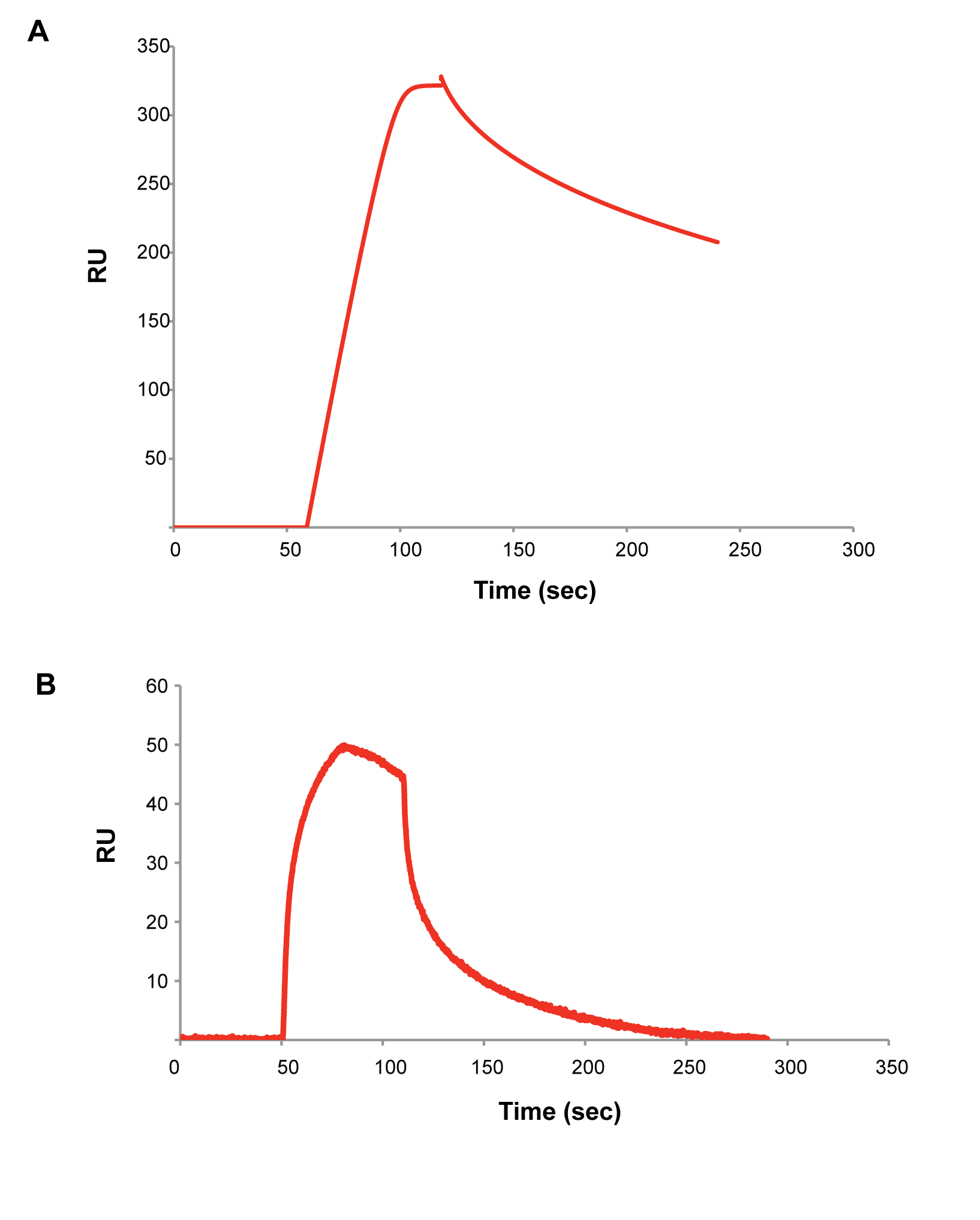

Supplement: Figure S2 — SPR sensorgram from injection of 100 nM synbody over A) immobilized AKT1 and B) immobilized GRP58. (0.35 MB TIF) [file pone.0010728.s005.tif]

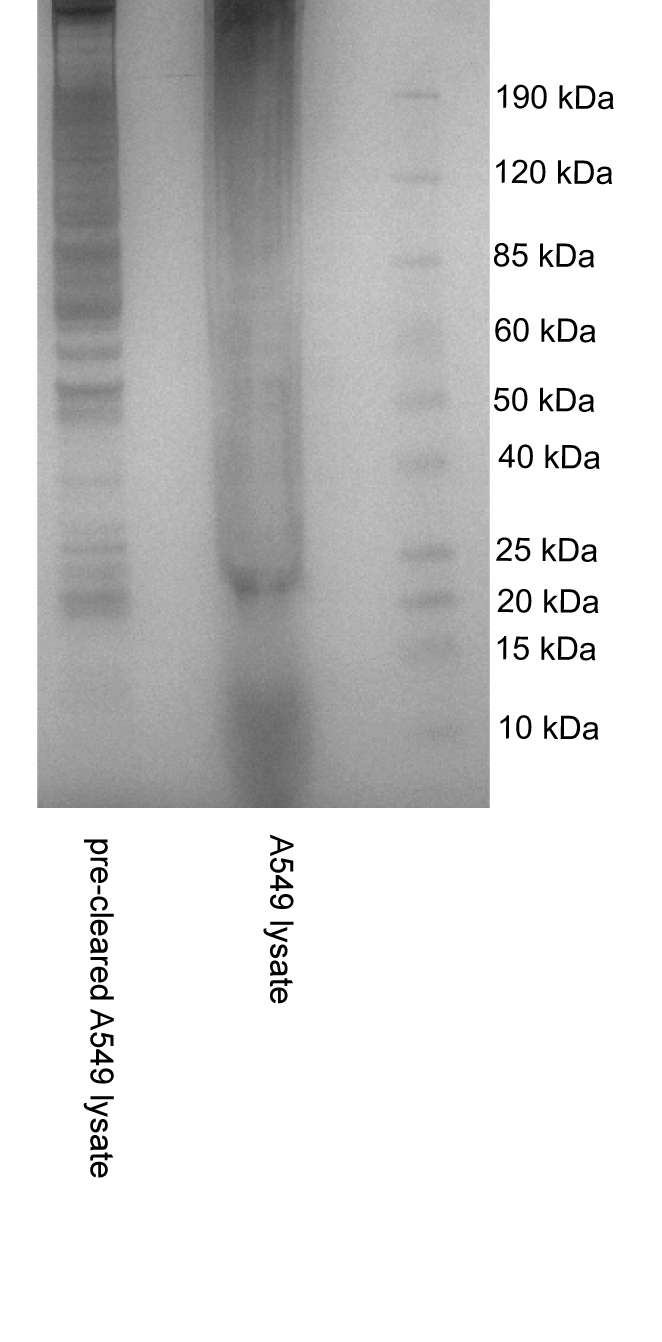

Supplement: Figure S3 — Silver stain gel of proteins present in A549 cell lysate that was pre-cleared using streptavidin coated magnetic beads and in un-cleared A549 cell lysate. (2.68 MB TIF) [file pone.0010728.s006.tif]

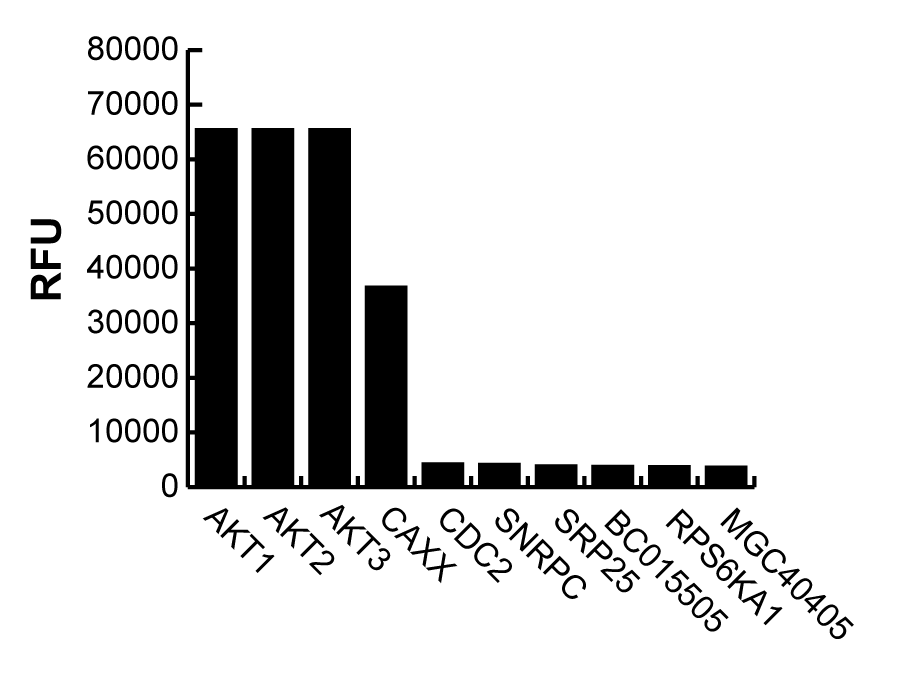

Supplement: Figure S4 — Top 10 proteins bound by anti-AKT1 monoclonal antibody on protein array. (0.09 MB TIF) [file pone.0010728.s007.tif]

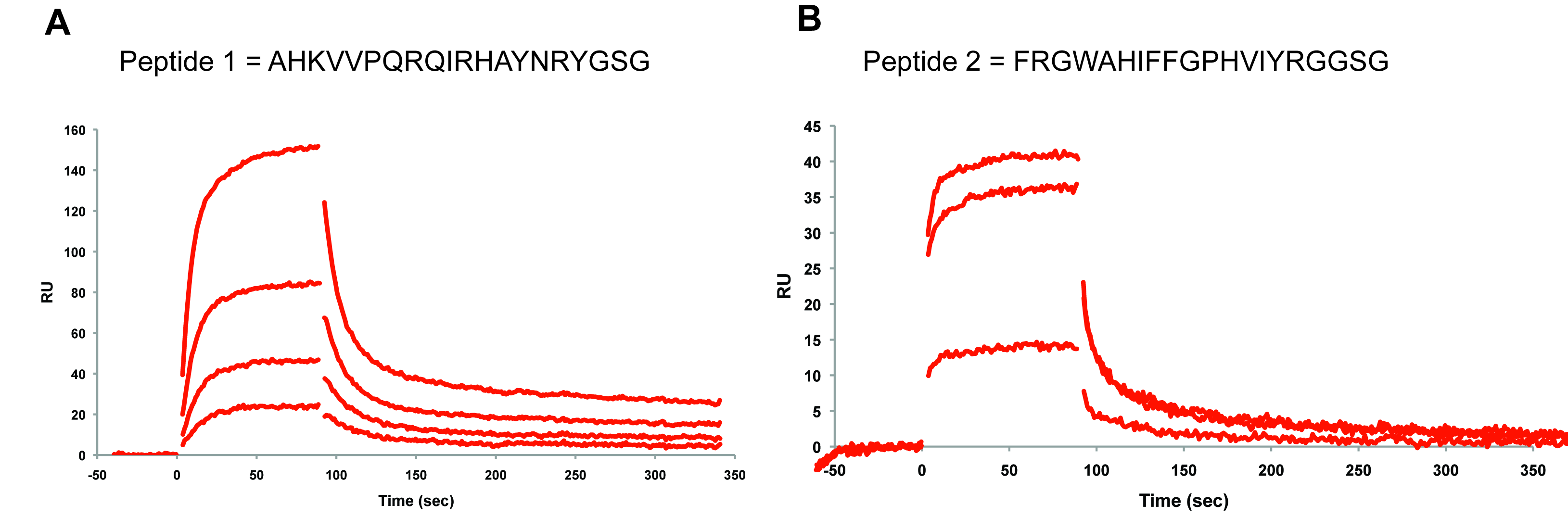

Supplement: Figure S5 — Sensorgrams from A) 1.88 µM, 0.94 µM, and 0.47 µM solutions of peptide 1 flowed over 15,584 RU of immoblized AKT1 and B) 20 µM, 10 µM, and 1 µM solutions of peptide 2 over 19,912 RU of immobilized AKT1. Dissociation constants were not determined due to the high immobilization levels of AKT1 used. (0.95 MB TIF) [file pone.0010728.s008.tif]

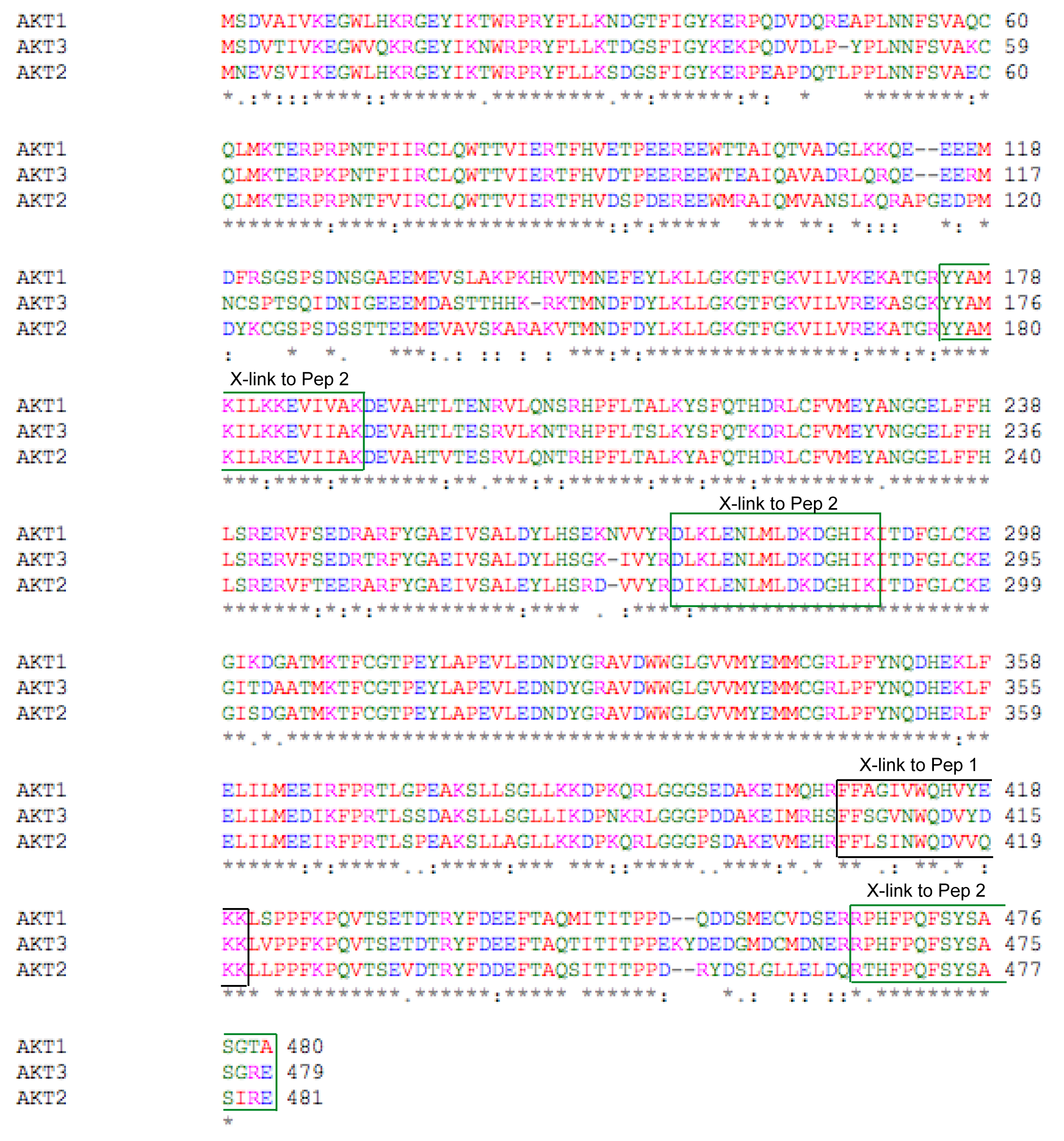

Supplement: Figure S6 — ClustalW alignment of AKT1, AKT2, and AKT3 illustrating AKT1 peptides identified in crosslinking experiments. (2.68 MB TIF) [file pone.0010728.s009.tif]

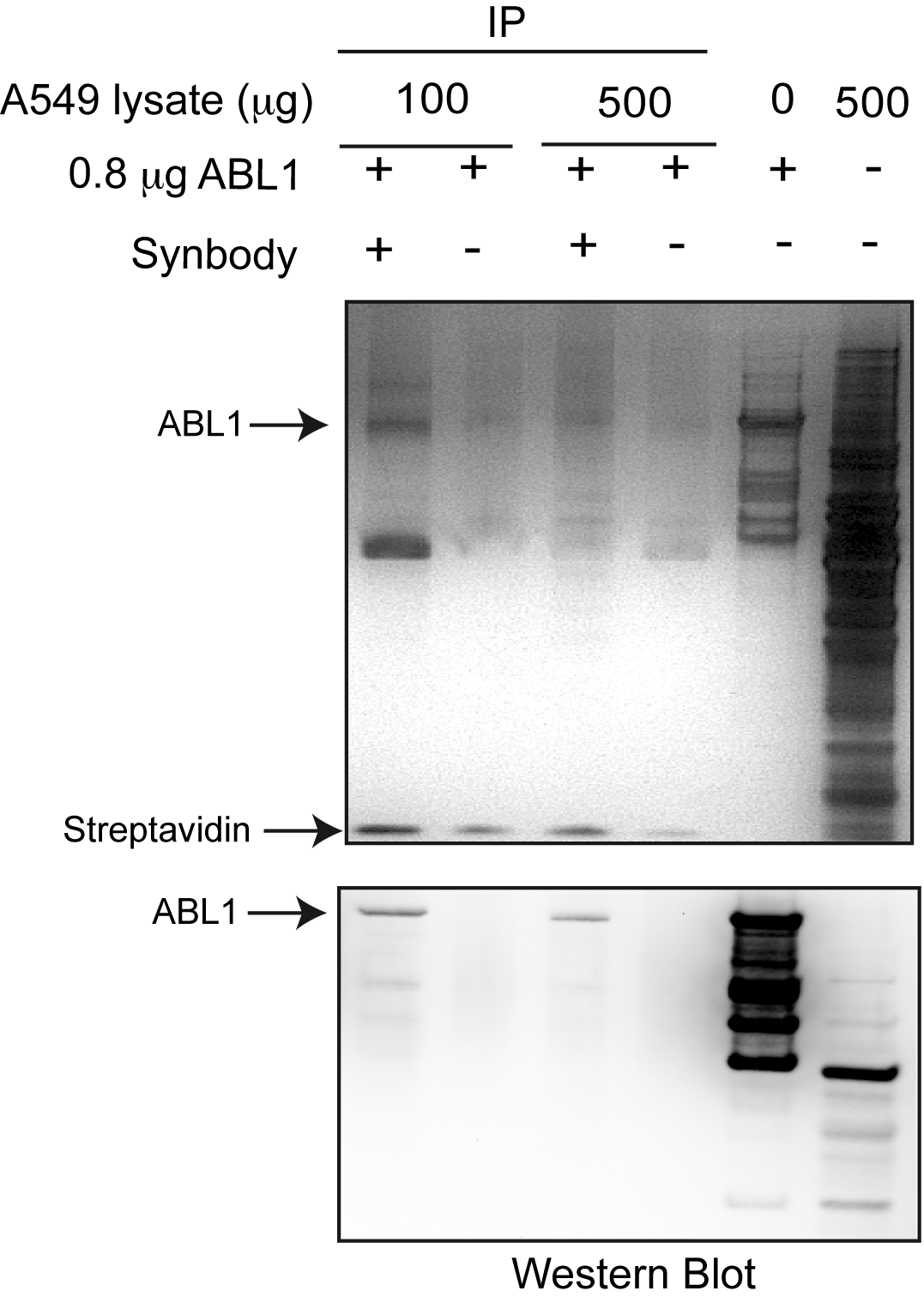

Supplement: Figure S10 — Silver stain of proteins precipitated by synbody 9 from solutions that contained 800 ng ABL1 spiked into either 100 or 500 µg of pre-cleared A549 cell lysate. Western Blot of same samples using a polyclonal anti-ABL1 antibody confirming the presence of ABL1. (4.93 MB TIF) [file pone.0010728.s013.tif]
